# Severe maternal morbidity in patients with pre-existing diseases: a prospective observational cohort study (ForMaT trial)

**DOI:** 10.1038/s41598-026-69115-z

**Published:** 2026-09-11

**Authors:** Philipp Kosian, Elena Jost, Chiara Flugel, Jennifer Nadal, Matthias Schmid, Waltraut M. Merz

**Affiliations:** 1 Department of Obstetrics and Prenatal Medicine, University Hospital Bonn, Venusberg Campus 1, 53127 Bonn, Germany; 2 Institute for Medical Biometry, Informatics, and Epidemiology, University Hospital Bonn, Venusberg Campus 1, 53127 Bonn, Germany

**Keywords:** Severe maternal morbidity, Pre-existing diseases, High-risk pregnancy, Antenatal complications, Diseases, Health care, Medical research, Risk factors

## Abstract

Severe maternal morbidity (SMM) is increasingly used as an indicator of maternal health. Registry-based studies have reported higher SMM rates among women with pre-existing diseases, but prospective data describing the timing and clinical context of these events remain limited. In this prospective observational cohort study conducted at a tertiary perinatal center in Germany between November 2022 and March 2024, SMM was defined according to the Centers for Disease Control and Prevention (CDC) criteria. Outcomes in women with pre-existing diseases were compared with two control groups: healthy women with obstetric risk factors and women with uncomplicated pregnancies. A total of 657 women with pre-existing diseases and 742 women in the control groups were included in the final analysis. Overall, 19 women experienced SMM, including 14 in the study group and 5 in the control groups. Women with pre-existing diseases had a higher risk of SMM than controls (relative risk (RR) 3.40, 95% confidence interval 1.22–9.49, adjusted for age, body mass index and parity). Half of the SMM events in the study group occurred antepartum and were attributable to the underlying disease, whereas events in the control groups occurred predominantly during the peripartum period and were mainly obstetric in origin. Women with pre-existing diseases also had higher risks of preterm birth (RR = 2.27; 95% CI 1.64–3.44) and neonatal intensive care unit admission (RR = 1.84; 95% CI 1.31–2.59). These findings suggest that pre-existing diseases substantially increase the risk of severe maternal morbidity and that a considerable proportion of events occur before delivery and are related to the underlying disease.

**Supplementary Information:**

The online version contains supplementary material available at https://doi.org/10.1038/s41598-026-69115-z.

## Introduction

Maternal complications can arise due to the underlying chronic disease, obstetric risk factors, or a combination of both, potentially leading to severe maternal morbidity (SMM)^1^. SMM comprises a spectrum of life-threatening diseases occurring during pregnancy, childbirth, or the postpartum period and is increasingly used alongside maternal mortality to assess maternal health^2–4^. SMM is also associated with adverse neonatal outcomes, including preterm birth, neonatal intensive care unit (NICU) admission, low birth weight, low Apgar scores, and perinatal mortality^5,6^. Contemporary surveillance reports and systematic reviews indicate that, in high-income countries, SMM occurs in roughly 6.7–11.4/1000 pregnancies including the postpartum period; nonetheless, published estimates range from 1 to 35/1000 pregnancies owing to differences in data sources, measurement criteria, and population risk factors^7^. Multiple definitions of SMM have been proposed, each shaped by its specific purpose and the type of data on which it relies: Global quality-improvement efforts (e.g., the World Health Organization (WHO) “maternal near-miss” tool) rely on bedside observations and basic labs to allow to audit individual cases^8^. Hospital quality programs such as ACOG/SMFM’s “screen-and-review” trigger in-depth case analysis and use simple, universally recorded events like ≥ 4 units of transfusion or an intensive care unit (ICU) stay^6^. U.S. public-health surveillance (Centers for Disease Control and Prevention’s (CDC) 21-indicator ICD algorithm) instead mines discharge codes so states can track trends at scale without chart review^9^. The CDC’s 21-indicator algorithm is now a widely used international metric for SMM because it relies exclusively on routinely collected ICD-10 data, demonstrates strong predictive validity for maternal death, and carries endorsements from leading professional bodies. Furthermore its publicly available code set facilitates straightforward implementation and enables cross-country comparison^9,10^.

Haemorrhage and hypertensive pregnancy disorders are common causes for SMM worldwide^11^, whereas in high-income settings rising SMM from chronic preexisting diseases layered on top of a managed—but still present—risk of obstetric complications is observed^12^. Over recent decades, the prevalence of maternal chronic diseases has increased in high-income countries, with rates rising from below 5% to over 25% in some regions^13–15^. Factors contributing to this trend include advanced maternal age, progress in the treatment of severe childhood diseases, and advances in assisted reproductive technologies^16–18^. While the overall SMM incidence in the general obstetric population is about 6.7–11.4/1000^7^ it rises steeply in women with pre-existing diseases, reaching up to 56/1000 births among multimorbid patients with three or more pre-exisiting diseases^10,19^.

Although the increased risk of SMM among women with pre-existing diseases has been documented in registry-based studies^7^, fewer prospective cohorts have examined the clinical context of these events^20^ or whether they are primarily related to the underlying disease or to obstetric complications at delivery. This prospective cohort study assessed SMM among women with pre-existing diseases using the CDC criteria^9^, evaluated the timing of these events, and compared the risk with two control groups of women with and without obstetric risk factors.

## Methods

### Study design

This study was an observational, prospective, longitudinal single-center cohort study. Recruitment and data collection started in November 2022 and lasted until March 2024, followed by a five-month follow-up period. It was part of a three-dimensional observational study (ForMaT). This study is reported in accordance with the Strengthening the Reporting of Observational Studies in Epidemiology (STROBE) guidelines^21^, was approved by the institutional ethics committee (341/22) and was conducted in accordance with the local legislation and institutional requirements. The participants provided their written informed consent to participate in this study.

The study protocol has been published previously^22^ and the study was registered in the German Clinical Trials Register (DRKS00030061) on the 28th of October 2022.

### Study population

Observation of participants covered the period from initial consultation in pregnancy to hospital discharge after birth. Assignment to study and control groups was applied during recruitment:

### Study group (S)

**Fig. 1:**
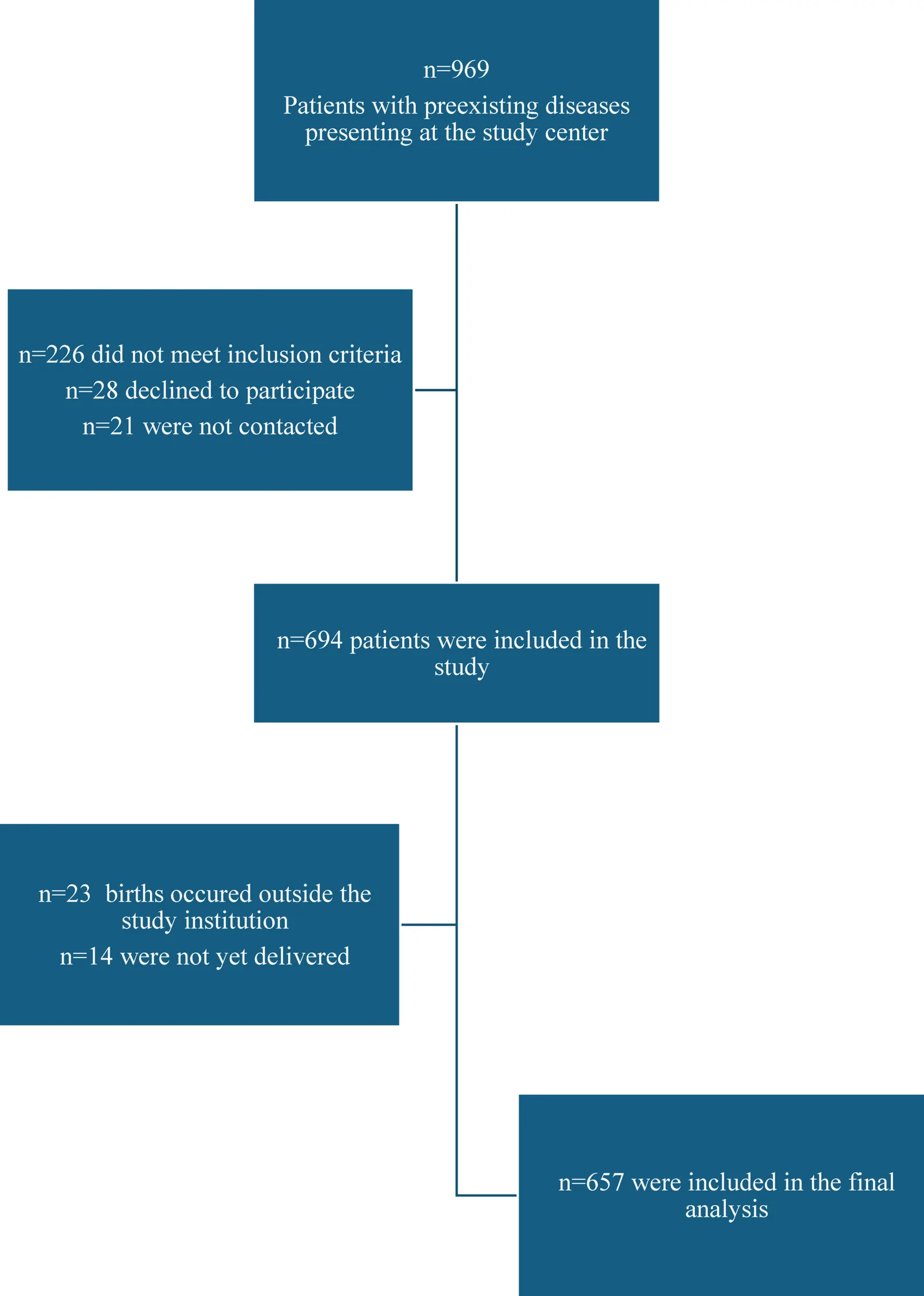
Patient selection flow chart for study group S.

The detailed flow of participant inclusion and exclusion is shown in Fig. 1.

For the purpose of this study, a pre-existing disease was defined as a chronic disease or long-term consequences of a previous medical event. Only diseases that could have an impact on pregnancy or delivery were included in the study (*n* = 657, 47%). Details and a full list of eligible and ineligible diseases is available in supplementary tables (sTables 1 and 2).

#### Control group (K1 and K2)

The group K1 consists of healthy pregnant women whose pregnancies were considered high-risk due to their obstetric history or complications during the current pregnancy (*n* = 483, 34.5%; 29 cases were excluded because the birth occurred outside the study center or the delivery had not yet taken place by the end of the observation period). Group K2 consists of healthy pregnant women carrying a singleton fetus in cephalic presentation who register for delivery at term following an uneventful pregnancy (*n* = 259, 18.5%; 53 cases were excluded because the birth occurred outside our institution or the delivery had not yet taken place by the end of the observation period). A full list of eligible obstetric diseases and complications is provided in a supplementary table (sTable 3).

#### Exclusion criteria for all groups

Women carrying higher-order multiple pregnancies (beyond twins) and those unable to fully understand the study’s purpose were not included. Additionally, healthy women referred to the study center due to fetal anomalies (e.g., aneuploidies or congenital malformations) were excluded. Participants were also excluded from the final analysis if delivery occurred outside our institution or had not yet taken place by the end of the follow-up period.

#### Assignment and stratification

Participants were allocated to the three study arms at the time of enrolment by the recruiting investigator, using a 2 : 2 : 1 ratio for groups S, K1, and K2.

#### Sample size

Sample size estimation was based on the primary medical outcome SMM and indicated that we needed to recruit 600 cases in the study group (S) and 900 controls (K1, K2). Typical confounders in studies of SMM—parity, multiple gestation, prior obstetric history, and pregnancy complications—were addressed explicitly through our recruitment strategy. Details are found in the published study protocol^22^.

Study data were collected and managed using REDCap electronic data-capture tools^23^ hosted at our institution. Data were retrieved from the hospital information system ORBIS (Dedalus Healthcare, Bonn, Germany) and ViewPoint™ (VGE HealthCare, Solingen, Germany).

#### Primary and secondary outcomes

Primary outcome was SMM according to the criteria by the CDC^9^, with the inclusion of maternal mortality and the transfusion of ≥ 4 blood products. Maternal diagnoses were coded using the German Modification of the International Classification of Diseases, 10th Revision (ICD-10-GM), and procedures were coded using the German Procedure Classification system (OPS). SMM was not identified using routinely generated administrative or billing data. Instead, during data collection, maternal diagnoses, procedures, and complications relevant to the CDC SMM definition were systematically recorded from the complete clinical record. The corresponding ICD-10-GM diagnosis codes and procedure codes required for the CDC SMM algorithm were manually assigned and entered into the study database for research purposes. Secondary outcomes included, among others, the following variables: Caesarean delivery (yes vs. no), Preterm birth (defined by gestational age (GA) at birth < 37 weeks), Neonatal intensive care (NICU) admission (yes vs. no) and length of stay after labour (grouped in ≤ 3 days and > 3 days).

#### Statistics

Continuous variables are presented as means ± standard deviations (SDs, normally distributed variables) or medians with interquartile ranges (IQRs, non-normally distributed variables); categorical variables are presented as frequencies and percentages.

To control for confounding and selection bias at baseline, a propensity score analysis was performed. The propensity score — calculated via logistic regression with baseline covariates maternal age, body mass index (BMI) before pregnancy, and parity — estimates the conditional probability of belonging to the study group S (women with pre-existing diseases).

To compare the primary and secondary outcomes in the study and control groups, inverse-propensity-score-weighted log-binomial regression models (IPW) with group (S vs. K1 + K2) as covariate were fitted to the data.

Associations are reported as relative risks (RR) with 95% confidence intervals (CI).

In an additional sensitivity analysis of the primary outcome, the study group S was compared to the control groups K1 and K2 separately (using the same log-binomial modeling approach as in the main analysis). All analyses were conducted using SAS 9.4 and R version 4.5.2.

## Results

A total of 1399 women were included in the study, distributed across three groups: 657 in the study group, 64.7% of those were multimorbid (defined as > 1 pre-existing disease), 483 in K1 and 259 in K2.

Baseline characteristics and outcomes are found in Tables 1 and 2. For each patient, the most clinically relevant pre-existing disease was prioritised and classified by organ system (Table 1). Furthermore, the appendix contains a version of Table 1 broken down by the groups S, K1 and K2 (sTable 4).

**Table 1:** Characteristics, study group (S) and control groups (K1 + K2).

Variable	Total *N* = 1399	Women with pre-existing diseases (S) *N* = 657 (47.0%)	Control Group (K1 + K2) *N* = 742 (53.0%)
Maternal age (years), mean ± SD	32.7 ± 5.0	32.4 ± 5.1	32.9 ± 4.9
< 18	3 (0.2)	2 (0.1)	1 (0.1)
≥ 18 - < 35	892 (63.8)	434 (31.1)	458 (32.7)
≥ 35	504 (36.0)	221 (15.8)	283 (20.2)
BMI before pregnancy (kg/m^2^), mean ± SD	26.1 ± 6.1	26.7 ± 6.5	25.1 ± 5.7
≤ 18.5	47 (3.4)	21 (1.5)	26 (1.9)
> 18.5 -≤24.9	691 (50.4)	292 (21.3)	399 (29.1)
> 25 - ≤ 30	321 (23.4)	153 (11.2)	168 (12.2)
> 30 - ≤ 35	194 (14.1)	97 (7.1]	97 (7.1)
> 35	119 (8.7)	71 (5.2)	48 (3.5)
Parity ≥ 1, N (%)	739 (52.8)	311 (22.2)	428 (30.6)
Tobacco use, N (%)	66 (4.8)	32 (2.3)	34 (2.5)
≥ 1 Medication, N (%)	570 (40.7)	323 (23.1)	247 (17.7)
Duration of care (weeks), median (min-max)	4.6 (0.4–32.9]	8.4 (0.4–32.9]	3.7 (0.4–25.1)
Disease characteristics of women with pre-existing diseases (S,* N *= 657)			
Main diagnosis, N (%)			
Autoimmune		133 (20.2)	
Cardiovascular/Kidney		141 (21.5)	
Other		383 (58.3)	
Any mental disorder, N (%)		77 (11.7)	
Disease duration (years), mean ± SD		11.4 ± 9.8	
Multimorbidity, N (%)		425 (64.7)	
Preconception stability, N (%)*		532 (81.0)	

BMI: Body Mass Index; GA: gestational age; NICU: neonatal intensive care unit; * no hospitalization and no medication change in the six months before conception.

**Table 2:** Primary and secondary outcomes, study group (S) and control groups (K1 + K2).

Outcomes	Total *N* = 1399	Women with pre-existing diseases (S) *N* = 657 (47.0%)	Control Group (K1 + K2) *N* = 742 (53.0%)
Severe maternal morbidity, N (per 1000 pregnancies)	19 (13.6)	14 (21.3)	5 (6.7)
Preterm birth GA < 37 + 0 weeks, N (%)	150 (11.0)	100 (7.3)	50 (3.7)
Caesarean delivery, N (%)	626 (45.7)	317 (23.1)	309 (22.6)
NICU admission, N (%)	141 (9.9)	92 (6.5)	49 (3.4)
Length of stay after labor > 3 days, N (%)	240 (17.8)	164 (12.1)	76 (5.6)

GA: gestational age, NICU: neonatal intensive care unit.

### Unadjusted analysis

Overall, SMM occurred in 19 women (13.6/1000). The incidence of SMM in the study group was 21.3/1000, whereas in the group of healthy women with high-risk pregnancies (K1) it was 8.3/1000 and in the group of healthy pregnant women carrying a single child (K2) it was 3.9/1000. In the combined group K1 + K2 it was 6.7/1000, resulting in an unadjusted RR of 3.18 (95%CI 1.15–8.73).

### IPW-adjusted analysis

Prior to IPW, the baseline variables showed larger standardised mean differences between the group S and the control groups (K1 + K2); with IPW, this difference was considerably reduced, so that the means of most variables were close to zero, indicating a good balance between groups S and K1 + K2 (sFigure 1 A). This was also evident in the propensity score distributions of the groups: without IPW, there was little overlap; with IPW, the distributions showed a higher overlap (sFigure 1B). Women in the study group had a higher risk of experiencing SMM than women in the control group (K1 + K2). The corresponding adjusted RR was 3.40 (95% CI 1.22–9.49).

Notably, in the control group all severe maternal complications occurred either intrapartum or post-operatively after CD. Without exception those were obstetric or post-caesarean complications and, apart from one case, consisted of postpartum haemorrhage requiring transfusion. By contrast, in the study group S 50% of severe complications arose antepartum, including one maternal death following a pregnancy termination performed because of the woman’s critical disease. Among the 14 SMM cases in the study group, 7 were primarily attributable to the participant’s preexisting disease. In 4 cases, obstetric complications were the immediate cause of SMM but were influenced by the underlying disease and the remaining 3 cases were solely due to obstetric complications.

More than half of all SMM events (11/19) were transfusion-related. In control groups K1 and K2, transfusion accounted for the vast majority of SMM cases (4/5), indicating that haemorrhage was the primary contributing factor. The study group showed a higher overall rate of SMM, with approximately half of the cases attributable to complications related to pre-existing diseases rather than to transfusion alone (Table 3).

**Table 3:** Severe maternal morbidity with and without transfusion, study group (S) and control groups (K1 + K2).

	*N*	SMM, *N* (per 1000 pregnancies)	SMM without transfusion, *N* (per 1000 pregnancies)
Overall	1399	19 (13.6)	9 (6.4)
S	657	14 (21.3)	8 (12.2)
K1 + K2	742	5 (6.7)	1 (1.3)
K1	483	4 (8.3)	1 (2.1)
K2	259	1 (3.9)	0(0)

SMM: severe maternal morbidity.

**Fig. 2:**
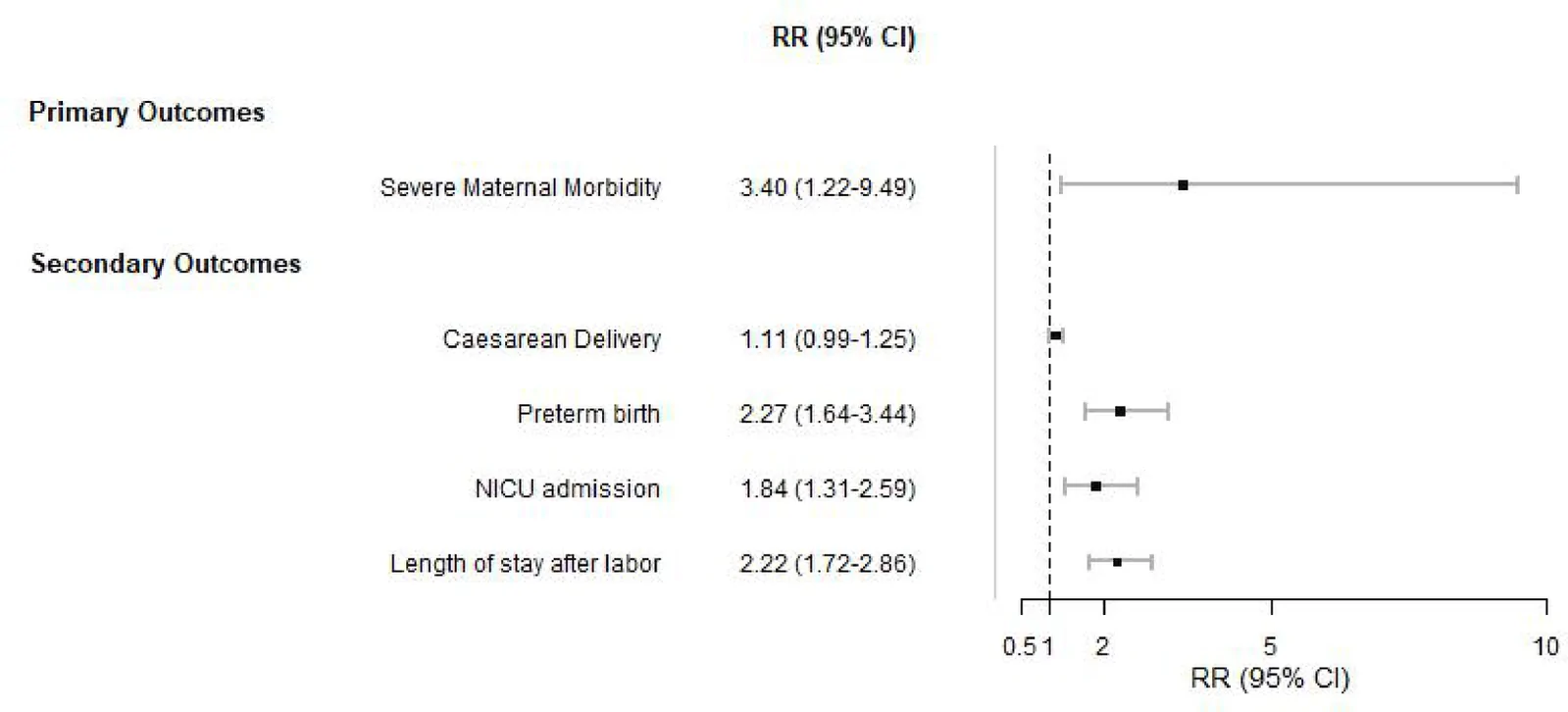
IPW adjusted analysis showing relative risks (95% CI) for the group factor (S vs. K1 + K2) associated with primary and secondary outcomes. Forest plot of IPW adjusted analyses. Relative risks (RR) with corresponding 95% confidence intervals are displayed on a logarithmic scale for the study group (S) and the different outcomes: SMM, preterm birth (< 37 weeks of gestation), NICU admission, CD and length of hospital stay for delivery. Dots represent point estimates of the RR; horizontal lines indicate 95% confidence intervals. The dashed vertical line at RR = 1 denotes the null effect. RR, relative risk; GA, gestational age; NICU, neonatal intensive care unit; CD, caesarean delivery; IPW, inverse probability weighting.

In the IPW adjusted analysis of the secondary outcomes, the study group S showed a higher risk for Caesarean delivery (RR = 1.11, 95%CI 0.99–1.25), preterm birth (RR = 2.27, 95%CI 1.64–3.14), NICU admission (RR = 1.84, 95%CI 1.31–2.59) and length of hospital stay after labour (RR = 2.22, 95%CI 1.72–2.86) (Fig. 2).

To assess whether the observed association was driven by the inclusion of low-risk pregnancies, a sensitivity analysis was performed comparing the study group (S) exclusively with women with obstetric risk factors (K1). Women with pre-existing diseases had an estimated higher risk for SMM (RR = 2.90). However, the 95% CI of this risk increase (0.96–8.90) included the value 1. The comparison of the study group (S) with group K2 showed an increased risk of 6.22 (0.81–47.60) for SMM.

## Discussion

In our prospective cohort study, women entering pregnancy with pre-existing diseases had a more than threefold higher risk of severe maternal morbidity (SMM) than healthy women with or without obstetric risk factors. Notably, half of all SMM events in this group occurred before delivery and were primarily related to the underlying maternal disease, whereas SMM in the control groups occurred predominantly during the peripartum period and was mainly obstetric in origin.

A recent systematic review by Henderson et al. (2025), encompassing studies published between 1993 and 2024, reported a median overall incidence of SMM between 6.7 and 11.4 per 1,000 pregnancies, including the postpartum period in one of the studies. Definitions of SMM varied substantially across studies. Those based on administrative data (32 definitions from 28 studies) had a median incidence of 11.4 per 1,000 pregnancies, with follow-up extending to up to 6 weeks postpartum, whereas definitions derived from primary medical records (13 definitions from 13 studies) had a median of 6.7 per 1,000 over the same follow-up period. Most definitions encompassed major organ dysfunction, severe morbidity categories, and critical interventions^7^. These findings align with our observed rate of 13.6 per 1,000 pregnancies, noting that follow-up ended at hospital discharge and did not extend through the full postpartum period.

Population-based data from high-income countries indicate that although SMM can occur in women classified as low risk, rates in this group are very low (4–5/1000) and mainly due to postpartum haemorrhage^24^, whereas pregnancies with obstetric risk factors have markedly higher rates^25^. Across diverse settings, population based studies from Canada^26^ and Denmark^27^ show that chronic diseases present before or early in pregnancy consistently drive SMM risk even more than peripartum events alone. Using California delivery discharges from 2016 to 2017, Leonard et al. (2020) demonstrated that a woman’s baseline health—captured by the cumulative weight of her pre-existing diseases— is a dominant determinant of SMM^28^. SMM affects about 10–30 per 1000 women with any pre-existing disease, but the risk rises with heavier comorbidity loads, reaching 100–190/1000 in the highest-risk strata^28^. This pattern is mirrored in our cohort, where the SMM incidence in the study group was 21.3/1000, a higher SMM rate than in the control groups. In 78.6% of cases, SMM was a direct or indirect consequence of a pre-existing disease.

An important aspect of our findings was the timing of events: 50% of SMM cases in women with pre-existing disease occurred before delivery and were related to the underlying disease. Although antepartum SMM has been described previously^20^, much of the available evidence is derived from retrospective population-based studies based on discharge data from delivery hospitalizations^7^. Our prospective data provide detailed clinical information on the timing of these events and show that, in medically complex pregnancies, SMM does occur before delivery and is not limited to the peripartum period.

In many SMM definitions, transfusion is a key component, accounting for 21–75% of cases when included, but its predictive value for true severe morbidity is limited, potentially inflating incidence by capturing less severe events^7^. Transfusing ≥ 4 units of blood usually is strongly associated with cardiac decompensation, ICU admission, and other organ-failure criteria^6^. By defining transfusion-related SMM as ≥ 4 units of blood products, we aimed to increase specificity for clinically significant events. In our study transfusion drives more than half of all SMM events. In control groups K1 + K2, almost all SMM cases are transfusion-related, making haemorrhage the dominant driver. By contrast, the study group showed a higher overall SMM rate, with approximately half of the events attributable to complications of the underlying diseases rather than to transfusion alone.

Our findings are consistent with the growing body of evidence demonstrating that women entering pregnancy with pre-existing diseases are at increased risk of adverse obstetric and neonatal outcomes. In a large population-based cohort including more than one million births, Brown et al. (2026) demonstrated that women with chronic medical conditions had increased risks of adverse perinatal outcomes, including preterm birth and severe neonatal morbidity or mortality^29^. Similarly, Admon et al. (2018) reported that women with multiple chronic conditions experienced higher rates of adverse obstetric outcomes, including cesarean delivery, as well as greater healthcare utilization during pregnancy and delivery^30^. In line with these observations, women with pre-existing diseases in our cohort had higher rates of cesarean delivery, preterm birth, and NICU admission than healthy controls, highlighting the substantial obstetric and neonatal burden associated with maternal chronic disease.

A major challenge in the field is the absence of a universally accepted definition of SMM. Published definitions vary widely in their conceptual scope and data source. This heterogeneity complicates cross-country comparisons, trend monitoring, and the evaluation of quality-improvement interventions^7^.

This study has limitations. It was conducted at a single tertiary care center, which may have resulted in a population with greater medical complexity than population-based cohorts, potentially limiting external validity. Second, the observation period did not encompass the full perinatal continuum; therefore, events occurring outside this interval may have been missed, potentially leading to an underestimation of the overall burden of SMM. This includes SMM events occurring within 42 days postpartum, which account for approximately 14–16% of all cases^3^. The low number of SMM events limits the precision of the effect estimates, as reflected by the wide confidence intervals. Thus, although our findings indicate an increased risk of SMM in women with underlying medical conditions, uncertainty remains regarding the magnitude of this association. A key strength of this study is its prospective design, which enables detailed, clinically grounded case analysis. In addition, the use of standardised CDC SMM criteria and explicit classification of transfusion thresholds enhances comparability with population-based surveillance data.

In conclusion, our study confirms that women with pre-existing diseases had an increased risk for SMM. Our findings further indicate that SMM in this population differs from that in healthy women, with a greater proportion of antepartum events related to the underlying disease. These results emphasize the importance of multidisciplinary antenatal care and improved risk stratification for women entering pregnancy with chronic disease.

## Supplementary Information

Below is the link to the electronic supplementary material.

Supplementary Material 1

Supplementary Material 2

Supplementary Material 3

## Data Availability

The datasets generated and analysed during the current study are available from the corresponding author on reasonable request.
