# Supplementary material for: Severe maternal morbidity in patients with pre-existing diseases: a prospective observational cohort study (ForMaT trial)
Source: Sci Rep. 2026 Sep 11;16:28486. doi: 10.1038/s41598-026-69115-z (PMC13569830; doi:10.1038/s41598-026-69115-z)
Supplement: Supplementary file 2 [file 41598_2026_69115_MOESM2_ESM.docx]

**STable 4: Characteristics study group (S) and per control group K1 and K2**

| **Variable** | **TOTAL**  **N = 1399** | **Women with**  **pre-existing diseases (S)**  **N = 657 (47.0%)** | **Control group (K1)**  **N = 483 (34.5%)** | **Control group (K2)**  **N = 259 (18.5%)** |
| --- | --- | --- | --- | --- |
| **Maternal age (years), mean ± SD** | 32.7 ± 5.0 | 32.4 ± 5.1 | 32.8 ± 4.9 | 33.0 ± 4.8 |
| < 18 | 3 (0.2) | 2 (0.1) | 0 (0) | 1 (0.1) |
| ≥ 18 - < 35 | 892 (63.8) | 434 (31.1) | 298 (21.3) | 160 (11.4) |
| ≥ 35 | 504 (36.0) | 221 (15.8) | 185 (13.2) | 98 (7.0) |
| **BMI before pregnancy (kg/m^2^), mean ± SD** | 26.1 ± 6.1 | 26.7 ± 6.5 | 26.6 ± 6.1 | 23.5 ± 4.1 |
| ≤ 18.5 | 47 (3.4) | 21 (1.5) | 16 (1.2) | 10 (0.7) |
| > 18.5 -≤24.9 | 691 (50.4) | 292 (21.3) | 215 (15.7) | 184 (13.4) |
| > 25 - ≤ 30 | 321 (23.4) | 153 (11.2) | 128 (9.3) | 40 (2.9) |
| > 30 - ≤ 35 | 194 (14.1) | 97 (7.1] | 75 (5.5) | 22 (1.6) |
| > 35 | 119 (8.7) | 71 (5.2) | 46 (3,4) | 2 (0.2) |
| **Parity ≥ 1, N (%)** | 739 (52.8) | 311 (22.2) | 300 (21.4) | 128 (9.1) |
| **Tobacco use, N (%)** | 66 (4.8) | 32 (2.3) | 28 (2.0) | 6 (0.4) |
| ≥1 Medication, N (%) | 570 (40.7) | 323 (23.1) | 181 (12.9) | 60 (4.3) |
| **Duration of care [weeks], median (min-max)** | 4.6 (0.4-32.9] | 8.4 (0.4 – 32.9) | 3.5 (0.4-25.1) | 4.0 (0.7-15.3) |

**Disease characteristics of women with pre-existing diseases (study group S, N = 657)**

| Main diagnosis |  |
| --- | --- |
| Autoimmune | 133 (20.2) |
| Cardiovascular/Kidney | 141 (21.5) |
| Other | 383 (58.3) |
| Any mental disorder, N (%) | 77 (11.7) |
| Disease duration (years), mean ± SD | 11.4 ± 9.8 |
| Multimorbidity, N (%) | 425 (64.7) |
| Preconception stability, N (%)* | 532 (81.0) |

BMI: Body Mass Index; GA: gestational age; ICU: Intensive Care unit; NICU: neonatal intensive care unit; * no hospitalization and no medication change in the six months before conception; •excluding admission for childbirth
