# Supplementary material for: Severe maternal morbidity in patients with pre-existing diseases: a prospective observational cohort study (ForMaT trial)
Source: Sci Rep. 2026 Sep 11;16:28486. doi: 10.1038/s41598-026-69115-z (PMC13569830; doi:10.1038/s41598-026-69115-z)
Supplement: Supplementary file 3 [file 41598_2026_69115_MOESM3_ESM.docx]

**SFigure 1: Covariate balance and propensity score distribution without and with Inverse Probability Weighting (IPW).**

(A) Love plot displaying absolute mean differences in covariates (BMI, parity, age) between comparison groups without and with IPW adjustment. Unadjusted values are shown as grey circles, whilst adjusted values are shown as black circles. The dashed vertical line indicates the threshold of 0.1 for acceptable covariate balance.

(B) Mirror histogram of propensity score distributions for groups K1+K2 (upper) and S (lower) without and with IPW adjustment. The y-axis represents absolute frequency and the sum of IPW weights, respectively.
